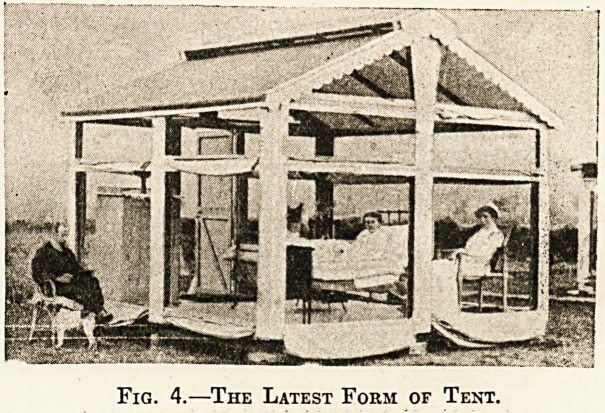# An Improved Form of Shelter for Use in the Open-Air Treatment of Phthisis

**Published:** 1912-01-20

**Authors:** H. Norman Marrett

**Affiliations:** Merivale Sanatorium, Sandon, Near Chelmsford.


### An Improved Form of Shelter for Use in the Open-Air Treatment of Phthisis.

By H. NORMAN MARRETT, M.R.C.S., L.R.C.P., Merivale Sanatorium, Sandon, near Chelmsford.

In most up-to-date sanatoria of the present day some form of shelter or veranda is in use. As an improvement upon this method of obtaining light and air, I have had tents constructed upon the following lines (see photographs). The roof, sides, and door are made entirely of canvas supported by a framework of wood. The floor is constructed of $\frac{3}{4}$-inch planks laid upon joists. For a light tent of this description the joists need not be of larger dimensions than 4 inches by 2 inches. To facilitate transport, I have found it convenient to make the boards of the floor in four sections, each of them being 6 feet square. The joists rest at their ends upon the framework of the tent, and are prevented from sagging in the middle by two or three bricks placed upon the ground. In this way the floor is raised some 12 inches from the ground, thus permitting a free current of air to circulate beneath the tent. Sufficient stability is given to the structure if the four corner posts are fixed from 2 to 3 feet into the ground. It is perhaps almost superfluous to add that the portions of the post buried in the ground should be previously tarred or, better still, creosoted. The various portions of the framework are held together by mortise and tenon joints. The canvas that forms the roof is nailed directly to the framework.

The arrangements of the sides of the tent are more complicated, as several essentials have to be borne in mind. Two of these are of great importance—namely, that it shall be possible to close separately any one of the openings, and that means shall be provided to prevent noise and draught

Fig. 1.—A General View of the Tents.

caused by flapping of the canvas. The first of these points can best be appreciated by a careful study of the accompanying photographs. The second desideratum is achieved by fixing the canvas in such a manner as to render it taut. As shown in the photographs, all skirting is avoided in the construction of these tents; there is, therefore, no possibility of the accumulation of dust. The tents are made 12 feet square; this is found to give ample room for the requirements of treatment. The roof is slanting, the highest part being 8 feet and the lowest part 3 feet 6 inches above the floor. The canvas forming the roof is treated in such a manner as to render it waterproof, but yet so as to enable

Fig. 2.—A Tent Wide Open, as it Should be Whenever Possible.

light to pass through it. This is best done by obtaining some ordinary linseed oil, boiling it without the addition of any other substance, and then filtering it through gauze so as to remove all gross impurities. The canvas shutters and blinds are raised or lowered as desired by a very simple arrangement of cords and pulleys. The canvas used is of such a mesh as to prevent the pores being closed when the canvas becomes wet with rain or snow. It is a curious fact that when the canvas is wet it becomes more transparent. It is thus seen that by these means the great object of surrounding the patient with the maximum amount of light and air is attained.

Now comes the question of draught. As has been already stated, this is a most important matter, and it plays no small part in influencing the progress of the cure. Wooden shelters are made to work either on a pivot or not. If worked on a pivot to avoid the wind, it is almost impossible to avoid a draught at certain times, as no matter how much care is taken in altering the position of the shelter it is very difficult to keep pace with the vagaries of the wind by day and quite impossible by night. Draughts cannot be avoided where verandas and fixed shelters are used. It is obvious that on some days, according to the wind, there may be no draughts either in a wooden shelter or on a veranda, but on other days a draught occurs which cannot be avoided.

In the case of canvas tents, on the other hand, neither the light nor the air can be excluded from them, and, further, if they are correctly located and manipulated it is impossible for the inmates to be in a draught. It is interesting to note that inter-catarrhal conditions and so-called "colds" do not occur with patients treated in tents as above described. The tent may be entirely closed, yet it will be still flooded with light. It may be kept open or it may be kept closed; in neither case can the air be excluded, nor can a draught be created, if, as I have said, the tents are properly manipulated. The position of the patient's bed, together with the canvas blinds and shutters, is, of course, varied according to the direction from which the wind or rain is coming. Fig. 3 shows

Fig. 3.—A Tent Partly Closed, Showing How the Patient May be Protected from Wind and Rain When Necessary.

a tent partly closed, illustrating how the patient can be protected from wind and rain when necessary.

A further advantage of this form of tent is that no fog or mist can enter it, no matter how foggy or misty the external atmosphere may be. Experience shows that, provided the nurse be instructed to close the shutter or shutters on the side or sides in the direction from which the wind is blowing, no fog or mist will enter the tent, notwithstanding that the rest of the tent remains wide open. The explanation of this fact is that the canvas acts as a filter to the air. The air passes through, but the particles of moisture are arrested. Air laden with moisture must be, and is, most detrimental to the patient. The walls of wooden shelters and so-called "open-air bungalows" may often be seen streaming with moisture. Scissors, penknives, keys, etc., cannot be kept in a wooden shelter without very soon showing signs of rust, whereas in a canvas tent they maintain their bright appearance for a considerable time. The main object in view is to have the tent kept as open as possible, both night and day.

The tents give rise to little or no inconvenience to the patient in the matter of cold. An extra blanket or two and a hot-water bottle are all that are required even in the severest weather to keep a patient comfortably warm. Apart from the occasions on which the shelter is closed to allow the patient to wash and to perform other needful functions, it ought never to be necessary to close more than two sides of the shelter at once, and then only on account of wind and rain.

With regard to furniture in the shelter. Besides the bed, only what is absolutely necessary for the comfort of the patient should be allowed, such as a lounge chair, writing-table, cupboard, and commode. The floor should be covered with linoleum of good quality. Rugs and other woollen materials are inadvisable.

A tent such as I have described above, with fittings complete, can be made for about £18 to £20. Fig. 4 shows a newer form of tent, which has a great many advantages over the sloping-roof tent, but is more expensive to build, costing £28. I have had very considerable experience of these canvas tents, having used them for over five and a half years in my sanatorium. Apart from the fact that cases treated by this tent system show far better results than those treated in wood or brick and mortar buildings, there is the enormous saving in expense where this particular tent system is employed. Not only are the results better, but the method is more economical. Although these tents were designed primarily with the object of treating phthisical patients, all tuberculous cases, both surgical and medical, do well in them. The tents are admirably adapted for patients wishing to carry out the open-air treatment in their own gardens.

In this brief article I have purposely confined myself to the question of fresh air, but I would add that no matter what system of "open-air treatment" is adopted, it is absolutely essential that it should be combined with unremitting care and attention to details, together with the immediate personal supervision of each individual patient, and that this is only possible where a very limited number of patients are treated at one and the same time.

With the prospect of a very large number of sanatoria being erected all over the country, under Mr. Lloyd George's scheme, the question of expense becomes a very important one. For a sanatorium such as I suggest large and costly buildings are avoided. All that is required is a house in the

Fig. 4.—The Latest Form of Tent.

country with a few acres of ground, which may be obtained at an annual cost of about £60 for rent, rates, and taxes. These places need little or no alteration, and are marketable assets, thus saving bricks and mortar. The best results, I feel sure, would be obtained if each sanatorium was limited to 50 patients. For a tent sanatorium to acommodate this number the annual cost need not exceed £3,000. The average cost per patient would be about 23s. per week.

## Figures and Tables

**Fig. 1. f1:**
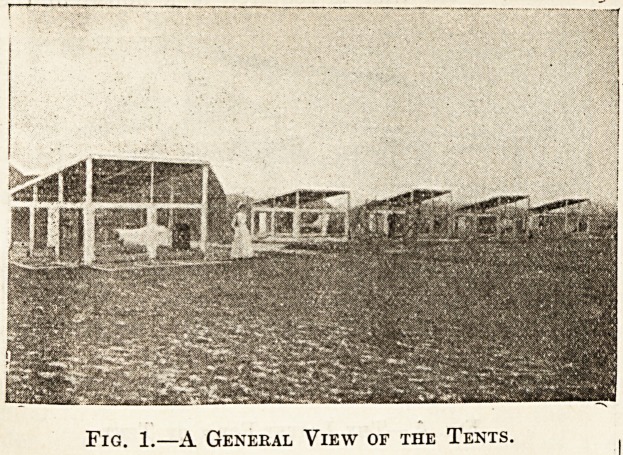


**Fig. 2. f2:**
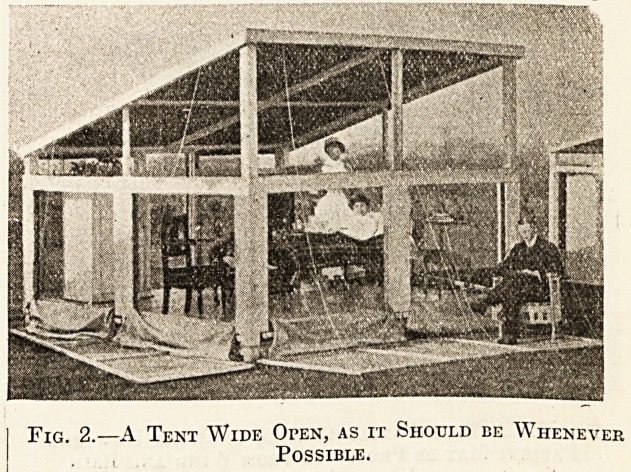


**Fig. 3. f3:**
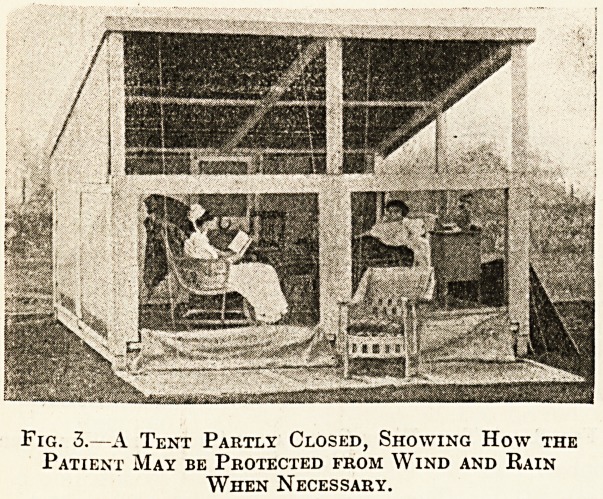


**Fig. 4. f4:**